# Conceptual, methodological, and measurement factors that disqualify use of measurement invariance techniques to detect informant discrepancies in youth mental health assessments

**DOI:** 10.3389/fpsyg.2022.931296

**Published:** 2022-08-02

**Authors:** Andres De Los Reyes, Fanita A. Tyrell, Ashley L. Watts, Gordon J. G. Asmundson

**Affiliations:** ^1^Comprehensive Assessment and Intervention Program, Department of Psychology, The University of Maryland at College Park, College Park, MD, United States; ^2^Resilient Adaptation Across Culture and Context Lab, Department of Psychology, The University of Maryland at College Park, College Park, MD, United States; ^3^Department of Psychological Sciences, University of Missouri, Columbia, MO, United States; ^4^Anxiety and Illness Behaviour Laboratory, Department of Psychology, University of Regina, Regina, SK, Canada

**Keywords:** Converging Operations, Diverging Operations, domain-relevant information, informant discrepancies, Operations Triad Model

## Abstract

On page 1 of his classic text, Millsap (2011) states, “Measurement invariance is built on the notion that a measuring device should function the same way across varied conditions, so long as those varied conditions are *irrelevant* [emphasis added] to the attribute being measured.” By construction, measurement invariance techniques require not only detecting varied conditions but also ruling out that these conditions inform our understanding of measured domains (i.e., conditions that do not contain *domain-relevant information*). In fact, measurement invariance techniques possess great utility when theory and research inform their application to specific, varied conditions (e.g., cultural, ethnic, or racial background of test respondents) that, if not detected, introduce measurement biases, and, thus, depress measurement validity (e.g., academic achievement and intelligence). Yet, we see emerging bodies of work where scholars have “put the cart before the horse” when it comes to measurement invariance, and they apply these techniques to varied conditions that, in fact, may reflect domain-relevant information. These bodies of work highlight a larger problem in measurement that likely cuts across many areas of scholarship. In one such area, youth mental health, researchers commonly encounter a set of conditions that nullify the use of measurement invariance, namely discrepancies between survey reports completed by multiple informants, such as parents, teachers, and youth themselves (i.e., *informant discrepancies*). In this paper, we provide an overview of conceptual, methodological, and measurement factors that should prevent researchers from applying measurement invariance techniques to detect informant discrepancies. Along the way, we cite evidence from the last 15 years indicating that informant discrepancies reflect domain-relevant information. We also apply this evidence to recent uses of measurement invariance techniques in youth mental health. Based on prior evidence, we highlight the implications of applying these techniques to multi-informant data, when the informant discrepancies observed within these data might reflect domain-relevant information. We close by calling for a moratorium on applying measurement invariance techniques to detect informant discrepancies in youth mental health assessments. In doing so, we describe how the state of the science would need to fundamentally “flip” to justify applying these techniques to detect informant discrepancies in this area of work.

## Introduction

What measurable sources of variance meaningfully inform our understanding of psychological phenomena? This question exemplifies a persistent struggle in scholarship on measurement, particularly when the phenomenon under investigation necessitates use of multivariate approaches to measurement. By construction, multivariate approaches result in researchers estimating *competing* sources of variance. One common approach involves leveraging multiple informants to assess a target person’s psychological functioning, such as self-reports from clients as well as reports from significant others in clients’ lives (e.g., caregivers and teachers in the case of youth; Hunsley and Mash, 2007). This approach requires differentiating variance that is shared among individual data sources in multivariate space (i.e., *common variance*) and variance that is particular to a given source (i.e., *unique variance*). Here, our central thesis is that much scholarship in measurement leads researchers down a path toward overly emphasizing common variance. This path even compels scholars to apply analytic techniques to measurement conditions that clearly violate assumptions underlying their use, and with the untoward effect of depressing measurement validity. In what follows, we suggest that measurement invariance techniques, particularly when they are incorrectly applied to scholarly work in specific areas (e.g., youth mental health), typify the concerns we raise. These concerns generalize to many other analytic techniques and, by extension, affect areas of study beyond the area highlighted in this paper (e.g., education, human development, pediatrics, personality, psychiatry, public health, sociology, and social work).

On page 1 of his classic text, Millsap (2011) presents a cogent operational definition of measurement invariance: “Measurement invariance is built on the notion that a measuring device should function the same way across varied conditions, so long as those varied conditions are *irrelevant* [emphasis added] to the attribute being measured.” Millsap’s definition serves as the “north star” of this paper. As the definition indicates, use of these techniques requires: (1) detecting conditions under which the measured domain might vary and (2) ruling out the possibility that these conditions inform our understanding of measured domains, or provide what we refer to as *domain-relevant information* (see also De Los Reyes et al., 2022a). This highlights a core principle: Efforts to distinguish between domain-relevant and domain-irrelevant measurement conditions should precede use of measurement invariance techniques.

Perhaps the clearest example of *proper* applications of measurement invariance techniques pertains to high-stakes testing scenarios, such as those found in academic and work settings (e.g., Meredith, 1993). In these settings, researchers have long-implemented standardized tests designed to index domains relevant to performance, such as academic achievement, scholarly aptitude, and intelligence (see Robertson and Smith, 2001; Reynolds et al., 2006). Selection decisions regarding placements in academic and/or work settings rely, in part, on the outcomes of these tests. Respondents who complete these tests also happen to be the applicants for placements in these settings, such as a law school applicant completing an entrance exam, or a workplace applicant completing a job-relevant task (e.g., of executive functioning). These tests produce individual differences in scores reflecting performance on setting-relevant domains, and respondents vary from each other on characteristics other than their test scores, such as demographic characteristics (e.g., age, gender identity, and racial/ethnic background). These sources of variance raise the question: *Should respondents’ demographic characteristics be considered when interpreting test scores?* Here, theory and research become particularly instrumental when deciding whether to apply measurement invariance techniques.

The consensus view among scholars is that variance in performance domains has little to do with respondents’ demographic characteristics (Council of National Psychological Associations for the Advancement of Ethnic Minority Interests [CNPAAEMI], 2016). In large part, this consensus view stems from the absence of a compelling theoretical and empirically based justification to claim otherwise (e.g., for a review relevant to gender differences and similarities in performance, see Hyde, 2014). This lack of compelling theory and evidence translates to researchers in the area of performance evaluations and decision-making leveraging measurement invariance techniques to great effect. In particular, measurement invariance techniques facilitate detecting items on performance tests that appear to operate differently as a function of respondents’ demographic characteristics (i.e., they exhibit differential item functioning; Osterlind and Everson, 2009). In these instances, group-specific response patterns reflect a kind of unique variance on test items, distinguishable from the variance that appears to share commonalities among groups or items that are *invariant* with respect to respondents’ demographic characteristics. If prior theory and research provided insufficient support for treating respondents’ demographic characteristics as domain-relevant information, then the group-specific, unique variance reflected by these characteristics reflects a *measurement confound*—an artifact of measurement that is irrelevant to the measured domain (e.g., domains linked to performance on an intelligence test). Here, items that function differently among respondents from different demographic groups reflect *biased* performance estimates. If such items were retained on a test, individual differences in respondents’ scores would be contaminated by measurement confounds. Retaining these biased items on a test and treating them the same as all other items−even those that are invariant concerning respondents’ demographic characteristics−would not only depress the validity of performance estimates but also render inaccurate any decisions based on these estimates (e.g., personnel selection, acceptance decisions).

High-stakes testing scenarios, such as those found in academic and work settings, comprise one area that exemplifies the proper use of measurement invariance. These scenarios highlight the consequences of not leveraging measurement invariance techniques when theory and research demand their use. However, the reverse is also true. The use of measurement invariance techniques when there are clear theoretical and empirical reasons to *avoid* using them poses similarly grave consequences to measurement validity and decision-making. Relatively underemphasized in psychological measurement is the study of circumstances in which researchers should refrain from using measurement invariance techniques.

In this paper, we address four aims. First, we briefly describe key measurement practices in youth mental health research, and the measurement conditions these practices often produce. Second, we describe a body of work that provides a compelling account of why researchers should avoid using measurement invariance techniques to detect common measurement conditions produced by youth mental health assessments. Third, we review recent uses of measurement invariance techniques in youth mental health and highlight the implications of (mis)applying measurement invariance techniques to youth mental health assessments. Fourth, we present a case for placing a moratorium on applying measurement invariance techniques to detect the common measurement conditions described in this paper. In doing so, we describe how the state of the science would need to fundamentally “flip” to justify applying measurement invariance techniques to detect common measurement conditions produced by youth mental health assessments.

## Understanding youth mental health requires multi-informant approaches to assessment

When considering how researchers use youth mental health assessments, one readily sees how *social context* factors into the domain-relevant information assessors obtain when using these assessments. This is because mental health domains are intimately connected to youths’ social environments. For instance, core and associated features of commonly diagnosed mental health conditions among youth tend to stem from how youth interact with their social environment. Examples of these features include fear and avoidance of unfamiliar social situations in social anxiety, inconsistent or harsh parenting in conduct disorder, maladaptive peer relations in attention-deficit/hyperactivity disorder (ADHD), and exposure to acute life stressors in major depression (American Psychiatric Association, 2013). Not all youth experience the same home environments, peer relations, school settings, and general life circumstances relevant to mental health functioning. In this respect, in youth mental health, “context” manifests as an individual differences variable, with considerable variation within and between youth undergoing evaluation. Two youth who experience ADHD may differ substantially in their clinical presentations. One youth’s symptoms may manifest largely at school, such as in peer-related difficulties and academic challenges, whereas another youth’s symptoms may manifest largely at home, due to inconsistent parenting, among other factors. In fact, context-relevant individual differences factor prominently in interventions designed to address youths’ mental health needs, for not only ADHD but all other domains for which youth receive mental health services.

Social context also factors into developing psychosocial interventions and interpreting their effects. These interventions consist of techniques (e.g., exposure, behavior modification, cognitive restructuring, social skills training) that require personalization, to “fit” the unique needs of clients and their social environments (see also Kazdin and Rotella, 2009; Weisz et al., 2012, 2015; Kazdin, 2013). In fact, we have long known that clients’ contexts vary, in part, because they each contain *contingencies* or factors that precipitate and/or maintain a client’s needs (see Skinner, 1953). To illustrate, the disciplinary methods parents use at home might differ from teachers’ methods at school, and social interactions youth encounter at school (e.g., teasing, bullying) might involve factors that elicit mental health concerns that are absent in other areas of a youth’s social environment (e.g., neighborhood near their home). In sum, context plays a key role in youth mental health research.

The importance of context necessitates leveraging context-sensitive modalities for measuring domains relevant to understanding youth mental health. The most common approach to collecting these context-sensitive data involves soliciting reports from multiple *informants* with expertise in observing youth in their naturalistic environment, notably in contexts like home and school (Hunsley and Mash, 2007). Within this *multi-informant approach*, researchers commonly collect reports from parents (i.e., to estimate behaviors displayed at home), teachers (i.e., to estimate behaviors displayed at school), and youth themselves (i.e., to estimate behaviors displayed across multiple contexts; see Kraemer et al., 2003; De Los Reyes and Makol, 2022a). Multi-informant assessments are commonplace in research about youth mental health services. For instance, users of the multi-informant approach apply it to index symptoms of mental health concerns (e.g., internalizing and externalizing domains; Achenbach, 2020) and associated features of these concerns (e.g., family functioning domains; De Los Reyes and Ohannessian, 2016), as well as psychosocial strengths (e.g., social skills; Gresham and Elliott, 2008; De Los Reyes et al., 2019a). By extension, the multi-informant approach features prominently in research germane to these domains. For example, data from multi-informant assessments largely comprise the foundation of estimates of intervention effects within the controlled trials literature (see Weisz et al., 2005), as well as the understanding of how domains of youth mental health concerns are related to one another (Achenbach, 2020), and associated features and risk factors of mental health concerns (De Los Reyes et al., 2015). Given the ubiquity of multi-informant assessment in youth mental health, researchers have long sought to understand the measurement conditions produced when leveraging this approach to assessment.

## Measurement invariance: The wrong “sentence” for multi-informant data derived from youth mental health assessments

In this section, we review one application of measurement invariance techniques in youth mental health, and demonstrate how little justification youth mental health researchers have for applying measurement invariance techniques to detect common measurement conditions observed with data from multi-informant assessments. In particular, we focus on the most commonly observed measurement condition in youth mental health. In this area, researchers often observe large discrepancies between informants’ reports of measured domains (i.e., *informant discrepancies*). A teacher might report a relatively higher degree of mental health concerns (e.g., disruptive behavior) in a student in their class, relative to estimates from the student’s parent(s). Discrepancies also manifest between reports taken from teachers and youth, as well as between youth and their parents.

Youth mental health researchers began observing these informant discrepancies decades ago (e.g., Lapouse and Monk, 1958). Decades later, hundreds of studies document that youth mental health assessments commonly produce informant discrepancies. For example, in a meta-analysis of 119 studies published between 1960 and 1986, Achenbach and colleagues (1987) estimated that, on average, informants’ reports of youth mental health domains (i.e., internalizing and externalizing concerns) correspond at low-to-moderate magnitudes (i.e., mean *r* = 0.28; Cohen, 1988). Nearly 30 years later, a meta-analysis of 341 studies published between 1989 and 2014 detected the exact same mean correspondence estimate observed by Achenbach and colleagues (i.e., *r* = 0.28; De Los Reyes et al., 2015). Further, a more recent cross-cultural meta-analysis revealed that this low-to-moderate level of correspondence manifests in multi-informant assessments of youth mental health conducted globally, as demonstrated by studies conducted in over 30 countries and every inhabited continent (De Los Reyes et al., 2019b). Additional characteristics of the meta-analytic literature support the robust nature of informant discrepancies. Low-to-moderate magnitudes of between-informant correspondence manifest when assessing concerns on the autism spectrum (Stratis and Lecavalier, 2015), associated features of mental health concerns (e.g., parenting, peer relations; Hawker and Boulton, 2000; Korelitz and Garber, 2016), and assessments of psychosocial strengths (e.g., social competence; Renk and Phares, 2004). Informant discrepancies also play an important role in meta-analytic estimates of intervention effects. Specifically, meta-analyses of controlled trials of psychosocial interventions for youth mental health reveal that intervention effects range from small-to-large in magnitude (e.g., Cohen’s (1988) *d* ranging from 0.3 to 0.8+), depending on the type of informant completing the outcome measure (see Casey and Berman, 1985; Weisz et al., 1987, 1995, 2006, 2017; De Los Reyes and Kazdin, 2006, 2008).

Informant discrepancies also manifest *within* the delivery of services, such that informants vary considerably in not only what general needs require services (e.g., targeting anxiety vs. mood vs. ADHD), but also the specific goals these services ought to address (e.g., improving a youth’s social skills with peers, decreasing parental use of inconsistent disciplinary strategies at home; De Los Reyes et al., 2022a). In terms of basic research, these discrepancies have enormous implications for detecting associated features and risk factors of mental health concerns, given that detections of such links also depend on the types of informants completing the measures of both mental health concerns and associated features/risk factors (see Hawker and Boulton, 2000; Youngstrom et al., 2003; De Los Reyes and Kazdin, 2005). Taken together, issues surrounding the application of measurement invariance techniques to data from multi-informant assessments are of paramount importance to all aspects of youth mental health research, because the varied measurement conditions reflected by informant discrepancies comprise the *defining characteristics* of assessments in this area.

### Conceptual foundations for interpreting informant discrepancies in youth mental health

If youth mental health assessments commonly produce informant discrepancies, then what might these discrepancies reflect? How clinicians and researchers go about multi-informant assessment has the “look and feel” of how journalists solicit sources for news stories. In an effort to gain a holistic understanding of a given topic, journalists don’t pick their sources at random. Rather, they strategically identify sources that reside at all corners or contours of the story topic. Youth mental health researchers similarly consult with informants who have *expertise* in observing youth in clinically relevant contexts. For instance, when researchers collect reports from teachers *and* parents, it is an acknowledgment that teachers have expertise in observing youth behavior within one set of social contexts and their constituent contingencies (i.e., school), whereas parents have expertise in observing behavior within an often fundamentally distinct set of contexts and contingencies (i.e., home). As noted in recent work on these issues (De Los Reyes et al., 2022a), the kinds of informants from whom youth mental health scholars collect reports are regarded as *structurally different* informants, or informants who systematically vary in the processes, through which they provide reports, *and* the processes bear some relevance to our understanding of the measured domain (see also Eid et al., 2008; Geiser et al., 2012; Konold and Cornell, 2015; Konold and Sanders, 2020).

**FIGURE 1:**
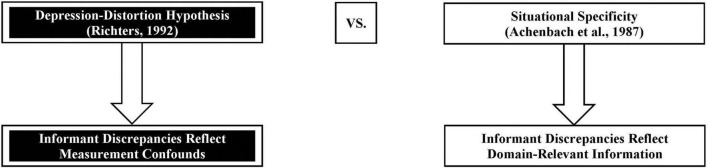
How theory and research about informant discrepancies ought to inform decision-making on when to apply measurement invariance techniques to detect informant discrepancies in youth mental health research. In research on multi-informant assessments of youth mental health, competing theories exist for what informant discrepancies reflect. One theory (i.e., depression-distortion hypothesis; Richters, 1992) claims that discrepancies reflect mood-congruent rater biases, such that a negative mood state compels an informant (e.g., parent) to attend to, encode, recall, and rate more negative youth behaviors, relative to informants who do not experience such mood states (e.g., teacher); accordingly, informant discrepancies reflect measurement confounds. In contrast, the other theory (i.e., situational specificity; Achenbach et al., 1987) claims that discrepancies reflect the notion that youth vary in the contexts in which they display mental health concerns, and the informants from whom assessors solicit reports (e.g., parents, teachers, and youth) vary in the contexts in which they observe youth; accordingly, informant discrepancies reflect domain-relevant information.

As mentioned previously, the decision to apply measurement invariance techniques requires careful attention to theory and research on the varied conditions that one seeks to detect (e.g., respondent characteristics and informant discrepancies). Given the structurally different nature of multi-informant approaches to the assessment of youth mental health, it is perhaps ironic that, when it comes to theory, empirical attention dedicated to what informant discrepancies reflect stems from the following *competing* theories: (a) the depression-distortion hypothesis (Richters, 1992) and (b) situational specificity (Achenbach et al., 1987). The former theory makes the claim that informant discrepancies reflect systematic rater biases (i.e., a measurement confound), whereas the latter makes the claim that informant discrepancies reflect, in part, domain-relevant information (Figure 1). We require data to reconcile these competing theories, and determine the appropriateness of applying measurement invariance techniques to detect informant discrepancies.

**FIGURE 2:**
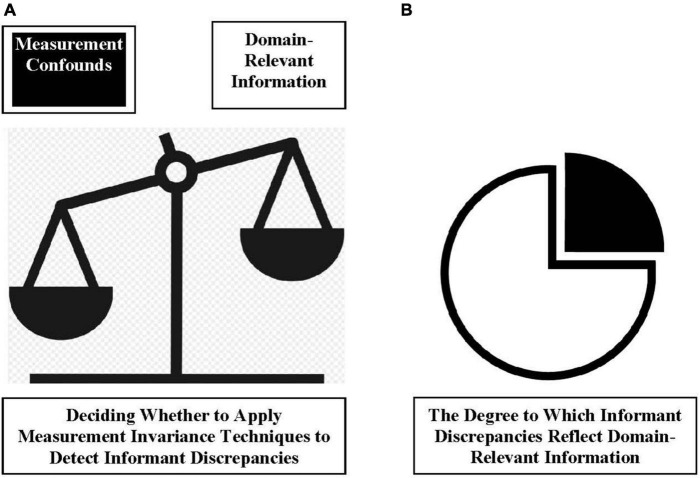
Graphical depiction of the discrete nature of decisions regarding the use of measurement invariance techniques to detect informant discrepancies in youth mental health research. Given the existence of competing theories about what informant discrepancies reflect (see Figure 1), one requires a “referee” to decide when to apply measurement invariance techniques. The only reasonable means by which to resolve the issue of competing theories is to put the theories “to the test” using empirical data produced by well-constructed validation studies. As **(A)** depicts, the definition offered by Millsap (2011) logically results in one deciding to use measurement invariance techniques only when empirical data (i.e., results from well-constructed validation studies) clearly tilt in favor of inferring that the measurement conditions one seeks to detect (e.g., informant discrepancies) reflect measurement confounds. Note that this is a distinct set of decisions from determining the degree to which informant discrepancies reflect domain-relevant information **(B)**. Indeed, both of these decisions ought to be grounded in empirical data linked to domain-relevant data conditions. Having said that, and whereas the decision to use measurement invariance techniques is discrete, determining the composition of variance in informant discrepancies is dimensional, because measures of all psychological domains likely reflect a “mix” of domain-relevant information and measurement confounds (i.e., no one measurement is “perfect”).

The decision to apply measurement invariance techniques is a discrete decision (Figure 2A). Recall Millsap’s (2011) definition is as follows: When a user applies measurement invariance techniques to detect a varied measurement condition, such as informant discrepancies, they are *required* to treat the detection of that condition as synonymous with detecting a measurement confound. Thus, to apply measurement invariance techniques to detect a given set of varied measurement conditions, a user needs the available evidence to tilt considerably in favor of measurement confounds as an explanation for the presence of these varied conditions. The discrete decision to apply measurement invariance techniques is distinct from estimating the degree to which informant discrepancies reflect domain-relevant information (Figure 2B). All instrumentation likely contains some degree of imprecision in measurement. Thus, even if a given measurement of informant discrepancies largely reflects domain-relevant information, it likely also contains some variance reflected by measurement confounds. A justified use of measurement invariance techniques requires that the data tilt so far in favor of measurement confounds as an explanation of a measurement condition (e.g., informant discrepancies), that optimizing measurement validity *necessitates* use of measurement invariance techniques.

In recent work, De Los Reyes and Makol (2022b) described the methodological limitations of prior work on the depression-distortion hypothesis. Due to the space constraints imposed by this article type (i.e., *Frontiers*’ Conceptual Analysis), an extended discussion of these limitations in the context of the use of measurement invariance techniques in youth mental health is presented elsewhere (see Online Supplementary Material). This allows us to dedicate our attention to research informed by situational specificity, which we posit as being most closely aligned with the principles and practices of multi-informant assessments in youth mental health, namely, the use of structurally different informants to assess youth.

Briefly, the notion of situational specificity holds that if youth inherently display individual differences in their social environments (i.e., not all youth have identical home or school environments), then the contingencies that elicit mental health concerns also vary within and across youth. By logical extension, some youth may live in social environments, whereby contingencies that elicit their concerns manifest in one context to a greater degree than other contexts (e.g., school > home and vice versa). In contrast, other youth may live in social environments whereby contingencies that elicit their concerns manifest similarly across contexts (i.e., school = home), and still, other youth live in social environments typified by contingencies that contribute to relatively healthy functioning across contexts. Structurally different informants vary in their *opportunities* to observe how youth behave within contexts and contingencies relevant to their mental health. Thus, discrepancies between these informants’ reports reflect, in part, the degree to which youth mental health varies across contexts.

**FIGURE 3:**
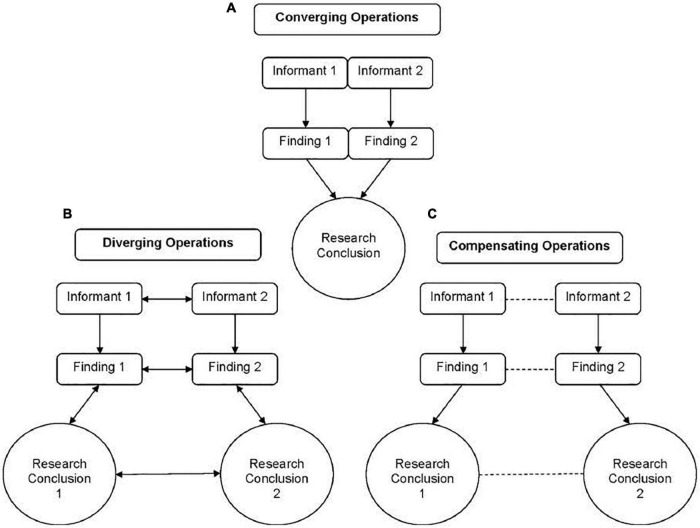
Graphical representation of the research concepts that comprise the Operations Triad Model. The top half **(A)** represents Converging Operations: a set of measurement conditions for interpreting patterns of findings based on the consistency within which findings yield similar conclusions. The bottom half denotes two circumstances, within which researchers identify discrepancies across empirical findings derived from multiple informants’ reports and, thus, discrepancies in the research conclusions drawn from these reports. On the left **(B)** is a graphical representation of Diverging Operations: a set of measurement conditions for interpreting patterns of inconsistent findings based on hypotheses about variations in the behavior(s) assessed. The solid lines linking informants’ reports, empirical findings derived from these reports, and conclusions based on empirical findings denote the systematic relations among these three study components. The dual arrowheads in the figure representing Diverging Operations convey the idea that one ties meaning to the discrepancies among empirical findings and research conclusions and, thus, how one interprets informants’ reports to vary as a function of variation in the behaviors being assessed. On the right **(C)** is a graphical representation of Compensating Operations: a set of measurement conditions for interpreting patterns of inconsistent findings based on methodological features of measures or informants. The dashed lines denote the lack of systematic relations among informants’ reports, empirical findings, and research conclusions. Originally published in De Los Reyes et al. (2013a). ©Annual Review of Clinical Psychology. Copyright 2012 Annual Reviews. All rights reserved. The Annual Reviews logo and other Annual Reviews products referenced herein are either registered trademarks or trademarks of Annual Reviews. All other marks are the property of their respective owner and/or licensor.

Situational specificity has important implications for understanding and interpreting multi-informant assessment outcomes. Figure 3 graphically depicts the dominant model used in youth mental health to interpret these assessment outcomes (i.e., the Operations Triad Model; De Los Reyes et al., 2013a). This model extends more traditional models for interpreting findings derived from multiple sources, namely, Converging Operations (Garner et al., 1956). In youth mental health, Converging Operations (Figure 3A) manifests when informants’ reports yield data that produce the same research findings (e.g., both reports support a youth client’s anxiety diagnosis, both reports support significant reductions in symptoms post-treatment). In fact, within Converging Operations anything less than converging findings reflects a measurement confound (e.g., random error, rater biases; Watts et al., 2022).

The meta-analytic work we described previously (e.g., Achenbach et al., 1987; De Los Reyes et al., 2015) indicates that in youth mental health research, Converging Operations rarely occur. This necessitates also conceptualizing informant discrepancies, and within the Operations Triad Model, not all informant discrepancies are created equally. To illustrate, informant discrepancies that reflect situationally specific manifestations of the measured domain represent Diverging Operations (Figure 3B), such that this form of informant discrepancy reflects domain-relevant information. In contrast, informant discrepancies that reflect measurement confounds harken back to Millsap’s (2011) operational definition of measurement invariance techniques, namely, varied measurement conditions that are irrelevant to the domains about which informants provide reports (e.g., random error and rater biases). These domain-*irrelevant* discrepancies represent Compensating Operations (Figure 3C).

In sum, multiple conceptual models facilitate use of multi-informant assessments and interpreting the nature of the informant discrepancies they often produce. Yet, it would be incorrect to assume that a given interpretation (e.g., that informant discrepancies reflect Diverging Operations) validly reflects informant discrepancies observed in any one youth mental health study. These conceptual models merely reflect a starting point when interpreting informant discrepancies. Indeed, these models ought to be *falsifiable*, such that they inform empirical tests of their veracity.

### How validation testing facilitates detecting the domain-relevant information in informant discrepancies

A key strength of situational specificity lies in its falsifiability. If situational specificity did not provide a thorough account of informant discrepancies, then well-constructed studies of this theory should reveal non-significant links between informant discrepancies and measured indicators of phenomena relevant to understanding youth mental health. Falsifiability allows us to directly test the question: *Do informant discrepancies contain data that inform our understanding of youth mental health?* This question bears a striking similarity to the questions that drive work on the score validity of measurement instruments in psychology (see Nunnally and Bernstein, 1994; Hinkin, 1995; Garb, 2003; Hunsley and Mash, 2007; Kazdin, 2017). In this respect, psychometric tests of score validity (e.g., construct, incremental, and criterion-related validation procedures) exemplify an ideal “fit” for empirically scrutinizing the concepts underlying the Operations Triad Model and, by extension, situational specificity. If the Operations Triad Model holds that not all cases of informant discrepancies are created equally, then well-constructed validation tests ought to facilitate distinguishing those informant discrepancies that reflect Diverging Operations from those that reflect Compensating Operations. By construction, the domain-relevant information reflected by Diverging Operations is uncorrelated with the domain-*irrelevant* measurement confounds reflected by Compensating Operations. Taken together, validation testing helps rule out the possibility that Compensating Operations characterize *all* informant discrepancies.

Over the last 15 years, numerous studies from multiple investigative teams have revealed various instances in which informant discrepancies likely contain domain-relevant information. The key to these studies has been the use of domain-relevant indicators as criterion measures in validation studies, such as ratings of observed behavior made by trained raters, scores from performance-based tasks, and readings from physiological instruments (for reviews, see De Los Reyes and Aldao, 2015; De Los Reyes et al., 2015, 2020). These criterion measures demonstrate *independence* in modalities, relative to informants’ reports. This independence in modalities allows researchers to rule out the possibility that any relations between informants’ reports and criterion measures arose simply because of *criterion contamination* or rater-specific variance, whereby both informants’ reports and validation criteria relied on the same measurement modalities to estimate youth functioning (see Garb, 2003).

Consider some validation tests focused on interpreting informant discrepancies. Recent meta-analyses show that parent and teacher reports of preschool children’s disruptive behavior display low-to-moderate levels of correspondence (Carneiro et al., 2021). Prior studies also highlight that parent-teacher dyads display considerable heterogeneity in patterns of reporting across developmental periods, as some dyads disagree in their reports, whereas other dyads agree (see Fergusson et al., 2009; Lerner et al., 2017; Sulik et al., 2017; Makol et al., 2021). In line with the notion of situational specificity, De Los Reyes et al. (2009) compared patterns of parent and teacher reports of disruptive behavior against independent assessments of children’s actual behavior (i.e., using the Disruptive Behavior Diagnostic Observation Schedule; Wakschlag et al., 2010). They found that when a teacher endorsed disruptive behavior that the parent did not (and vice versa), it signaled context-specific displays in actual behavior. De Los Reyes et al. (2009) leveraged validation testing to rule out the possibility that Compensating Operations explained all patterns of informant discrepancies observed in the sample of parent and teacher reports they examined. This is because informant discrepancies contained domain-relevant information, in the form of data pointing to the specific contexts in which children displayed disruptive behavior.

Other evidence supports the Diverging Operations interpretation of informant discrepancies within the context of predictive validity, such as the prediction of youth mental health outcomes. For example, in a sample of adolescents receiving an intake assessment for internalizing concerns within an acute care setting, Makol et al. (2019) discovered the same structure of parent-adolescent reporting patterns described previously with parent-teacher patterns, with some dyads disagreeing in their reports of internalizing concerns, whereas other dyads agreeing. Patterns of parent-adolescent agreement and disagreement observed at the intake assessment predicted key treatment characteristics during adolescents’ stays in acute care. Notably, adolescents, for whom parents reported relatively high internalizing concerns that were not corroborated by the self-reports of the adolescents, were at particularly high risk of being administered intensive treatment regimens, including standing antipsychotics and locked-door seclusion. These findings accord with work demonstrating that informant discrepancies predict other domain-relevant criteria, including treatment outcomes for youth anxiety (Becker-Haimes et al., 2018; Zilcha-Mano et al., 2021), treatment outcomes for youth trauma (Humphreys et al., 2017), pulmonary function in pediatric asthma assessments (Al Ghriwati et al., 2018), history of mental health service use (Makol and Polo, 2018), observed behavior within school- and home-based tasks (De Los Reyes et al., 2022b), and a host of youth psychosocial outcomes both concurrently and longitudinally in studies of community samples (e.g., internalizing/externalizing concerns, substance use, and risky sexual behaviors; Laird and De Los Reyes, 2013; Lippold et al., 2013, 2014; Ohannessian and De Los Reyes, 2014; Human et al., 2016; Laird and LaFleur, 2016; Nelemans et al., 2016).

In light of the evidence (for more extensive reviews, see De Los Reyes et al., 2019a,b, 2020, 2022a; De Los Reyes and Makol, 2022a,b), one key notion warrants comment. As noted in Figure 2B, none of the evidence rules out the possibility that at least some informant discrepancies reflect measurement confounds, like rater biases. However, the key decision point here—*Whether or not to apply measurement invariance techniques to detect informant discrepancies in youth mental health assessments*—is not a “Figure 2B” decision, but rather a “Figure 2A” decision. Regarding the decision point on whether to use measurement invariance techniques, the balance of theory and evidence tilts more toward informant discrepancies reflecting domain-relevant information rather than measurement confounds. The state of the science on these issues should be appreciated in much the same way as interpreting the evidence when carrying out any other kind of clinical or research activity. The evidence-based intervention literature would guide selecting an intervention for use in clinical work or research that *has* evidence of its potential utility in addressing clients’ needs, over an intervention with no such evidentiary support (see also Chambless and Ollendick, 2001; Kazdin, 2017; Weisz et al., 2017). The evidence-based assessment literature follows similar principles (see Hunsley and Mash, 2007). In the case of informant discrepancies in youth mental health research, both theory and the *preponderance* of the evidence points to a greater likelihood that any given instance of informant discrepancies reflects domain-relevant information vs. measurement confounds. This means that youth mental health researchers retain greater levels of measurement validity in scores taken from multi-informant assessment data if they refrain from applying measurement invariance techniques to detect informant discrepancies.

### How the domain-relevant information in informant discrepancies disqualifies “sentencing” these discrepancies to tests of measurement invariance

To truly understand how the state of the science on informant discrepancies disqualifies one from applying measurement invariance techniques to detect informant discrepancies in youth mental health assessments, it might be useful to think of the science as evidentiary exhibits in a legal proceeding. In Table 1, we frame the state of the science in just this way. When deciding on the verdict of a civil matter in the courts of common law countries, judges instruct juries to consider the *preponderance of the evidence* in favor of one of two opposing sides (i.e., plaintiff vs. defendant; Demougin and Fluet, 2006). Where does the majority of the evidence lie: On the side of the plaintiff or defendant? As with civil cases, here we have two opposing sides (i.e., competing theories; see Figure 1), and, thus, the question is as follows:

**TABLE 1:** Pieces of evidence (i.e., exhibits) that point toward informant discrepancies in youth mental health assessments as cases of domain-relevant information.

Exhibit	Description	Citation support
A	The notion of situational specificity	Achenbach et al., 1987
B	Youth mental health researchers rely on reports completed by structurally different informants	Kraemer et al., 2003; Eid et al., 2008; De Los Reyes and Makol, 2022a
C	Structurally different informants tend to complete parallel instruments that hold crucial measurement properties constant (e.g., item content, scaling, response options), thus reducing the likelihood that random error variance explains informant discrepancies	Hunsley and Mash, 2007
D	Several decades of research consistently point to large discrepancies between structurally different informants’ reports	De Los Reyes et al., 2015
E	Multi-informant assessments conducted across the globe consistently reveal discrepancies between structurally different informants’ reports	De Los Reyes et al., 2019b
F	Researchers observe discrepancies between structurally different informants’ reports, regardless of the instruments used to collect these reports and how well-established they might be	Achenbach and Rescorla, 2001; Gresham and Elliott, 2008
G	The best, most high-quality studies available to understand what informant discrepancies might reflect tend to show that these discrepancies reflect domain-relevant factors	De Los Reyes et al., 2022a
H	In tests of criterion-related validity that use independent assessments as criterion variables (i.e., observed behavior), approaches that meaningfully integrate data from structurally different informants’ reports outperform approaches that assume informant discrepancies reflect measurement confounds	Makol et al., 2020, 2021; De Los Reyes et al., 2022b
I	Any evidence of links between rater characteristics and informant discrepancies can be parsimoniously explained by domain-relevant factors	De Los Reyes and Makol, 2022b
J	Informant discrepancies tend not to be moderated by demographic characteristics of the youth being rated	Achenbach et al., 1987; De Los Reyes et al., 2015, 2019b

*Does the preponderance of the evidence point to informant discrepancies in youth mental health assessments reflecting domain-relevant information or measurement confounds?*

As seen in the exhibits presented in Table 1, the case here is, as lawyers often say, “open and shut.” Several of the exhibits presented in Table 1 (i.e., Exhibits A, B, D, E, and G), all of which were discussed in detail above, support the notion that informant discrepancies are more likely to contain domain-relevant information than be completely explained by measurement confounds.

### Recent examples of measurement invariance studies

The utility of framing the science on informant discrepancies as evidentiary exhibits in a legal proceeding is exemplified by another key component of case law, namely *precedent* (Schauer, 1987). In legal circles, precedent *compels* stakeholders within the proceeding to make decisions that are informed by verdicts rendered in previous cases. Our task here does not involve judging the merits of a legal case; yet, like precedent in legal proceedings, the operational definition of measurement invariance techniques advanced by Millsap (2011) ought to compel researchers to base their decisions on whether to use these techniques with strict adherence to prior theory and evidence.

Along these lines, Table 2 illustrates this phenomenon as it relates to prior measurement invariance studies. In Table 2, we list 12 recent measurement invariance studies. In the notes of Table 2, we provide a list of nearly 40 publications relevant to the aims and scope of the 12 studies listed in the table. For each of the 12 studies, we link, from among the list of nearly 40 publications, prior work that *preceded* the publication of each of the 12 studies by at least one calendar year. In each of these cases, one can find at least two publications that, at minimum, provided theoretical or empirical justification for the authors *refraining* from applying measurement invariance techniques to detect informant discrepancies in the multi-informant data used to address their study aims. Stated another way, whatever compelling evidence was available in the literature at the time the authors of each of these studies published their work, the case where available evidence was made pointed toward informant discrepancies reflecting domain-relevant information. This evidence should have compelled the authors to refrain from applying measurement invariance techniques to detect informant discrepancies in their data.

**TABLE 2:** Examples of measurement invariance studies focused on informant discrepancies in assessments of domains relevant to understanding youth mental health.

Authors (Year)	Article title	Citations as precedents^a^
Alvarez et al., 2020	Measuring perceptions of the therapeutic alliance in individual, family, and group therapy from a systemic perspective: Structural validity of the SOFTA-s	Fjermestad et al., 2016; De Los Reyes et al., 2019b
Bauer et al., 2013	A trifactor model for integrating ratings across multiple informants	Achenbach et al., 1987; Richters, 1992; Jouriles and Thompson, 1993; Kraemer et al., 2003; De Los Reyes et al., 2009; Fergusson et al., 2009; De Los Reyes, 2011
Curran et al., 2021	Psychometric models for scoring multiple reporter assessments: Applications to integrative data analysis in prevention science and beyond	Achenbach et al., 1987; Richters, 1992; Jouriles and Thompson, 1993; Kraemer et al., 2003; De Los Reyes et al., 2009, 2013a, 2019b,c,d,^2015^; 2019b; Fergusson et al., 2009; De Los Reyes, 2011; Hartley et al., 2011; Dirks et al., 2012; Laird and De Los Reyes, 2013; Lippold et al., 2013, 2014; Ohannessian and De Los Reyes, 2014; De Los Reyes and Ohannessian, 2016; Human et al., 2016; Laird and LaFleur, 2016; Nelemans et al., 2016; Ohannessian et al., 2016; Humphreys et al., 2017; Lerner et al., 2017; Sulik et al., 2017; Al Ghriwati et al., 2018; Becker-Haimes et al., 2018; Deros et al., 2018; Makol and Polo, 2018; Glenn et al., 2019; Makol et al., 2019
Florean et al., 2022	Measurement invariance of Alabama Parenting Questionnaire Across age, gender, clinical status, and informant	De Los Reyes et al., 2013c,d, 2019b; Laird and De Los Reyes, 2013; Lippold et al., 2013, 2014; Ohannessian and De Los Reyes, 2014; De Los Reyes and Ohannessian, 2016; Human et al., 2016; Laird and LaFleur, 2016; Nelemans et al., 2016; Ohannessian et al., 2016; Zilcha-Mano et al., 2021
Howe et al., 2019	Evaluating construct equivalence of youth depression measures across multiple measures and multiple studies	Achenbach et al., 1987; Kraemer et al., 2003; De Los Reyes et al., 2009, 2013a,2019b,^2015^; Fergusson et al., 2009; De Los Reyes, 2011; Hartley et al., 2011; Dirks et al., 2012; Humphreys et al., 2017; Sulik et al., 2017; Al Ghriwati et al., 2018; Becker-Haimes et al., 2018; Makol and Polo, 2018
Murray et al., 2021	Teacher vs. parent informant measurement invariance of the Strengths and Difficulties Questionnaire	Achenbach et al., 1987; De Los Reyes et al., 2009, 2013a; De Los Reyes and Aldao, 2015; Glenn et al., 2019; Fergusson et al., 2009; De Los Reyes, 2011; Hartley et al., 2011; Dirks et al., 2012; Lerner et al., 2017; Sulik et al., 2017
Olino et al., 2020	Evaluating maternal psychopathology biases in reports of child temperament: An investigation of measurement invariance	Achenbach et al., 1987; Richters, 1992; Jouriles and Thompson, 1993; Kraemer et al., 2003; De Los Reyes et al., 2009, 2013a, 2019b,c,d, 2015, 2019b; Fergusson et al., 2009; De Los Reyes, 2011; Hartley et al., 2011; Dirks et al., 2012; Laird and De Los Reyes, 2013; Lippold et al., 2013, 2014; Ohannessian and De Los Reyes, 2014; De Los Reyes and Ohannessian, 2016; Human et al., 2016; Laird and LaFleur, 2016; Nelemans et al., 2016; Ohannessian et al., 2016; Humphreys et al., 2017; Lerner et al., 2017; Sulik et al., 2017; Al Ghriwati et al., 2018; Becker-Haimes et al., 2018; Deros et al., 2018; Makol and Polo, 2018; Glenn et al., 2019; Makol et al., 2019
Olino et al., 2018	Is parent–child disagreement on child anxiety explained by differences in measurement properties? An examination of measurement invariance across informants and time	Achenbach et al., 1987; Richters, 1992; Jouriles and Thompson, 1993; Kraemer et al., 2003; De Los Reyes et al., 2009, 2013a, 2019b,c,d, 2015; Fergusson et al., 2009; De Los Reyes, 2011; Hartley et al., 2011; Dirks et al., 2012; Laird and De Los Reyes, 2013; Lippold et al., 2013, 2014; Ohannessian and De Los Reyes, 2014; De Los Reyes and Ohannessian, 2016; Human et al., 2016; Laird and LaFleur, 2016; Nelemans et al., 2016; Ohannessian et al., 2016; Humphreys et al., 2017; Lerner et al., 2017; Sulik et al., 2017
Russell et al., 2016	Agreement in youth-parent perceptions of parenting behaviors: A case for testing measurement invariance in reporter discrepancy research	De Los Reyes et al., 2013c,d; Laird and De Los Reyes, 2013; Lippold et al., 2013, 2014; Ohannessian and De Los Reyes, 2014; Laird and LaFleur, 2016
Schiltz et al., 2021	A psychometric analysis of the Social Anxiety Scale for Adolescents among youth with autism spectrum disorder: caregiver–adolescent agreement, factor structure, and validity	Achenbach et al., 1987; Kraemer et al., 2003; De Los Reyes et al., 2009, 2013a,2019b,^2015^, 2019b; De Los Reyes, 2011; Dirks et al., 2012; Humphreys et al., 2017; Al Ghriwati et al., 2018; Becker-Haimes et al., 2018; Deros et al., 2018; Makol and Polo, 2018; Glenn et al., 2019; Makol et al., 2019, 2020
Tagliabue et al., 2021	Latent congruence model to investigate similarity and accuracy in family members’ perception: The challenge of cross-national and cross-informant measurement (non) invariance	De Los Reyes et al., 2013c,d, 2019b; Laird and De Los Reyes, 2013; Lippold et al., 2013, 2014; Ohannessian and De Los Reyes, 2014; De Los Reyes and Ohannessian, 2016; Human et al., 2016; Laird and LaFleur, 2016; Nelemans et al., 2016; Ohannessian et al., 2016
Vanwoerden et al., 2022	Are we thinking about the same disorder? A trifactor model approach to understand parents’ and their adolescents’ reports of borderline personality pathology	Achenbach et al., 1987; Kraemer et al., 2003; De Los Reyes, 2011; Dirks et al., 2012; De Los Reyes et al., 2013a,c,d, 2015, 2019b; Laird and De Los Reyes, 2013; Lippold et al., 2013, 2014; Ohannessian and De Los Reyes, 2014; De Los Reyes and Ohannessian, 2016; Human et al., 2016; Laird and LaFleur, 2016; Nelemans et al., 2016; Ohannessian et al., 2016; Humphreys et al., 2017; Al Ghriwati et al., 2018; Becker-Haimes et al., 2018; Deros et al., 2018; Makol and Polo, 2018; Glenn et al., 2019; Makol et al., 2019, 2020; Baumgartner et al., 2021; Zilcha-Mano et al., 2021

^a^Citations note publications that pre-dated the publication year of the article, and that served as precedents to guide researchers toward refraining from applying measurement invariance techniques to study informant discrepancies.

## Implications for research and theory

### The measurement invariance oath

As yet, we have withheld discussion of a key element of measurement invariance studies—justification for use of measurement invariance techniques to address study aims. Measurement invariance studies about informant discrepancies in youth mental health assessments, such as those listed in Table 2, typically provide justifications for using measurement invariance techniques in the introduction sections of the papers reporting study findings. Still, the justifications researchers have often used (a) reflect a misunderstanding of the operational definition of measurement invariance (i.e., to detect domain-irrelevant measurement conditions; Meredith, 1993; Osterlind and Everson, 2009; Millsap, 2011) and (b) omit discussions of crucial evidence that would lean against the use of these techniques. Current standards for allowable justifications are insufficient because they allow authors to commit errors of commission and omission when considering the literature.

The central thesis of our paper is that youth mental health researchers need a new standard for applying measurement invariance techniques and, more importantly, a standard for *refraining* from applying these techniques. The standard we propose draws from two sources. The first pertains to a key element of Millsap’s (2011) definition of measurement invariance techniques, namely, applying these techniques only when theory and evidence deem a specific set of varied measurement conditions (e.g., as those indicated by discrepancies between informants’ reports of youth mental health) as *irrelevant* to measured domains. The second draws from seminal work by Lilienfeld (2007) on using evidence to detect psychosocial interventions that might result in harmful effects. As justification for identifying such interventions, Lilienfeld called attention to a core element of *The Hippocratic Oath*, namely, to first, do no harm. In particular, Lilienfeld applied this oath to understanding intervention effects in psychology, and to set a clear “low bar” for detecting interventions that *might* result in harm to the clients who receive them.

Assessment is the “evidence” in evidence-based interventions. Whatever standard we place on the quality of interventions we must also place on the quality of the evidence used to estimate intervention effects. As mentioned previously, multi-informant assessments comprise foundational pieces of evidence for many evidence-based interventions (see Weisz et al., 2005). If use of measurement invariance techniques potentially produces harmful effects when applied to data conditions that violate assumptions underlying their use (e.g., depress measurement validity, decrease the accuracy of clinical decision-making), then we must exhibit heightened sensitivity to using them.

Consequently, we advance a notion that is a “mashup” of sorts between the thinking of Millsap (2011) and Lilienfeld (2007)−*The Measurement Invariance Oath: First, do no harm to measurement validity.* By setting this low bar for refraining from use of measurement invariance techniques, the onus is put on the user of these techniques to provide strong evidence in support of their use but, also, evidence in support of the specific circumstances characterizing the intended use. Indeed, to do otherwise could have untoward effects on clinical decision-making. Consider a case in which a user applied measurement invariance techniques to a set of multi-informant assessments, for which the informant discrepancies they produce reflect domain-relevant information. In particular, consider the clinical implications of using these techniques to, for instance, remove items that exhibited differential functioning across informants. If the reason for this differential item functioning is domain-relevant (e.g., they index contextual variability in the behaviors being assessed), then clinicians that subsequently use these same assessments would be ill-equipped to detect the very kinds of context-specific displays of clinically relevant behaviors that necessitate use of multi-informant assessments (i.e., situational specificity). Losing this valuable feature of multi-informant assessments may have downstream effects on all elements of service delivery that are informed by these assessments (e.g., diagnosis, treatment planning, outcome assessments; see also De Los Reyes et al., 2022a).

### How the theory and evidence need to tilt to justify the use of measurement invariance techniques to detect informant discrepancies in youth mental health assessments

With the preponderance of the evidence pointing away from informant discrepancies reflecting measurement confounds in youth mental health assessments (Table 1), the use of measurement invariance techniques ought to require extraordinary evidence. This raises the question: *What kind of extraordinary evidence would justify the use of measurement invariance techniques to detect informant discrepancies in youth mental health assessments?* To address this question, we must highlight two ironies of work on informant discrepancies in youth mental health assessments.

The first irony is that the depression-distortion hypothesis has driven much of the work on what informant discrepancies reflect (see Richters, 1992; De Los Reyes and Kazdin, 2005; De Los Reyes and Makol, 2022b). Consider the nature of such a scholarly process. Most studies testing what informant discrepancies reflect have focused on the degree to which multi-informant assessments are “plagued” by rater biases, driven in large part by the observation of informant discrepancies. Yet, at the core of constructing multi-informant procedures is the notion that each informant’s report contributes useful information. A key “signal” of such utility would presumably be each informant’s report yielding data that other informants’ reports do not yield (see also De Los Reyes et al., 2015). In essence, this focus on rater biases took an observation (i.e., informant discrepancies) that should have been tested as a psychometric property of multi-informant assessments, and, instead, treated it as an inherent flaw. This process is akin to developing a depression instrument, and then, using the formative studies to focus on how “bad” the instrument is at assessing depression, as opposed to whether the scores it produced yield accurate estimates of depression. The second irony is that research on the depression-distortion hypothesis led informant discrepancies to work down the path we find ourselves, one for which youth mental health researchers currently have no justification for being on (i.e., applying analytic procedures to multi-informant assessments of youth mental health that equate informant discrepancies with rater biases).

**FIGURE 4:**
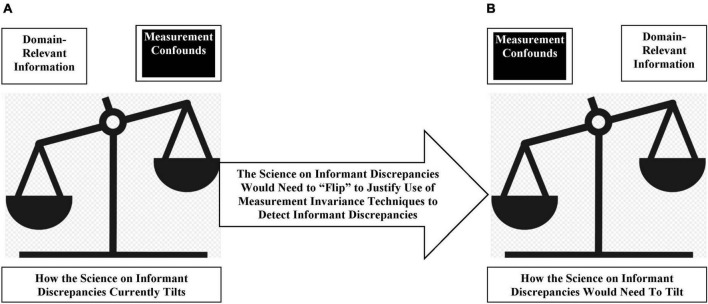
How the state of the science on informant discrepancies in youth mental health assessments would need to tilt to justify the use of measurement invariance techniques to detect informant discrepancies. **(A)** Depicts the current state of the science on these discrepancies, which clearly tilts in favor of informant discrepancies more likely reflecting domain-relevant information rather than measurement confounds. To justify the use of the measurement invariance techniques to detect informant discrepancies in youth mental health assessments would require theory and evidence that renders informant discrepancies synonymous with measurement confounds. This would require a complete “flip” of the theoretical and evidentiary bases of informant discrepancies as they manifest in youth mental health assessments **(B)**, such that the state of the science would need to clearly tilt in favor of the likelihood that informant discrepancies in youth mental health assessments reflect measurement confounds.

We opened this section with a question about the kind of evidence required to justify the use of measurement invariance techniques to detect informant discrepancies in youth mental health assessments. The answer to this question is that the state of the science would need to fundamentally “flip” to provide such a justification. We noted that Table 1 shows the current state of theory and evidence in this area of work points to informant discrepancies containing domain-relevant information. In Figure 4A, we graphically depict the state of the science, such that it tilts away from where the science would need to be to justify the use of measurement invariance techniques (i.e., toward measurement confounds).

For researchers to reach a justification for using measurement invariance techniques to detect informant discrepancies, they would have to do two things. First, they would have to detect as-of-yet undiscovered flaws in the body of theory and evidence reviewed in this paper relevant to the notion of situational specificity—flaws that render work testing the domain-relevant information contained in informant discrepancies as uninterpretable. Second, they would have to conduct studies testing for the presence of rater biases in informant discrepancies that correct for the limitations we noted in prior work on the depression-distortion hypothesis (see Online Supplementary Material). Future work on the depression-distortion hypothesis would have to contend with the reality that the *proposed* source of measurement confounds in this theory (i.e., informants’ levels of depression) contain, at minimum, a “mix” of domain-relevant variance and variance reflecting measurement confounds (see also Watts et al., 2022). However, all rater bias studies, to date, have made no attempt to deconstruct the variance in the purported source of rater biases (e.g., parent’s levels of depressive symptoms) into domain-relevant and domain-*irrelevant* components. In absence of this deconstruction, it is reasonable to assume that all prior work purporting to demonstrate a link between informants’ mood with mood-related rater biases might simply be reifying an effect that is already widely known in the literature, namely, that parents’ functioning is intimately connected to youth functioning (see Goodman and Gotlib, 1999).

### Procedures for addressing informant discrepancies in youth mental health assessments

To the degree that the primary goal of techniques like measurement invariance is to optimize measurement validity (i.e., by accounting for a variance that reflects measurement confounds), it is important to understand how the use of these techniques attempts to achieve that goal. Measurement invariance techniques equate sources of unique variance like informant discrepancies with the presence of measurement confounds. It logically follows that measurement invariance techniques emphasize common variance, namely, a focus on detecting variables (e.g., items on multi-informant surveys) that function invariantly across informants’ reports. Measurement invariance techniques are not alone in emphasizing common variance. The majority of analytic techniques currently used by researchers seeking to integrate or model multi-informant data in youth mental health emphasize common variance and treat unique variance as error, including composite scores (e.g., Martel et al., 2021), many applications of structural equations modeling (e.g., Eid et al., 2008; Bauer et al., 2013; Howe et al., 2019; Watts et al., 2022), and combinational algorithms (e.g., AND rule; Baumgartner et al., 2021).

In many respects, the theory and evidence reviewed in this paper apply generally to any analytic technique, for which an assumption underlying its use is that the only variance worth considering is common variance. When the data conditions violate this assumption (see Table 1 and Figure 4A), the use of such a technique will logically result in depressing measurement validity and, by extension, reducing statistical power to test hypotheses of interest (see also Markon et al., 2011).

Taken together, we see a need for a reimagining of how youth mental health researchers integrate multi-informant data. In particular, researchers who take multi-informant approaches to assess youth mental health must leverage integrative techniques that retain both common variance *and* domain-relevant unique variance. Although such techniques are in short supply; some exist and with documented support of their validity when applied to multi-informant data in youth mental health research (see Tables 1, 2).

For instance, when applied to multi-informant data (e.g., Lerner et al., 2017; Makol et al., 2019), person-centered models, such as latent class analysis (Bartholomew et al., 2002) allow for the detection of instances in which informants’ reports yield similar findings (i.e., Converging Operations; Figure 3A) and domain-relevant discrepant findings (i.e., Diverging Operations; Figure 3B), particularly when these patterns of findings can be examined in relation to domain-relevant criterion variables (e.g., observed behavior; for a review, see De Los Reyes et al., 2019a). Similarly, when examined as predictors of youth outcomes, polynomial regression (Edwards, 1994) allows for detecting variance explained by each informant’s report, as well as their interaction (i.e., patterns of agreement and disagreement between informants’ reports), in the prediction of domain-relevant criterion variables (for reviews, see De Los Reyes and Ohannessian, 2016; Laird, 2020). A third example of techniques that retain both common variance and domain-relevant unique variance can be found in factor-analytic work by Kraemer et al. (2003), who developed a form of principal components analysis that synthesizes multi-informant data into a common variance component and domain-relevant, unique variance components. A recent study indicates that this approach predicts independent assessments of observed behavior well above the predictive power of not only individual informants’ reports but also the average of reports (i.e., a composite score of reports; Makol et al., 2020) and sophisticated structural models that emphasize common variance (Makol, 2021). We encourage future work seeking to extend the portfolio of available integrative techniques that allow for considerations of common variance and domain-relevant unique variance, beyond those we briefly discussed.

## A call for a moratorium on using measurement invariance techniques to detect informant discrepancies in youth mental health assessments

To close this paper, we return to what we previously referred to as our “north star,” the operational definition of measurement invariance techniques advanced by Millsap (2011):

*“Measurement invariance is built on the notion that a measuring device should function the same way across varied conditions, so long as those varied conditions are irrelevant to the attribute being measured.”*

To arrive at a justification for using measurement invariance techniques, one must be justified in treating the detection of a varied condition (i.e., informant discrepancies) as synonymous with detecting a measurement confound (e.g., rater biases). Such a justification requires strict adherence to both theory and evidence. We demonstrated that in youth mental health research, there are competing theories regarding what informant discrepancies reflect (Figure 1). This necessitates the use of data to “settle the score” as to these competing theories (Figure 2). This also raises the possibility that theory and evidence will indicate that one should *avoid* the use of measurement invariance techniques to detect informant discrepancies (Figure 4A). However, at least one other implication of the definition bears emphasizing—the *goal* of using measurement invariance techniques. The goal of using measurement invariance techniques should be *laser-focused* on optimizing the psychometric rigor of scores taken from measurement instruments, namely, efforts that result in tangible, significant improvements to measurement validity. If the state of the science is not tilted toward equating informant discrepancies with the presence of measurement confounds (i.e., Figure 4B), the implication is clear: One optimizes measurement validity by avoiding the use of measurement invariance techniques to detect informant discrepancies.

Another element of the operational definition advanced by Millsap (2011) is that one requires *real evidence* to justify the use of measurement invariance techniques. In youth mental health research, the evidence overwhelmingly points away from informant discrepancies reflecting the kinds of measurement confounds that would justify the use of measurement invariance techniques. Rather, this evidence points toward the notion that informant discrepancies likely contain significant sources of domain-relevant information, data that directly pertain to the very youth mental health domains about which informants provide reports (Table 1). One cannot conduct a measurement invariance study in youth mental health research if they properly attend to this evidence, although many recent studies did not consider this wealth of prior evidence (Table 2).

Along these lines, might the issues raised in this paper apply to other measurement conditions in youth mental health? As previously mentioned, when interpreting the outcomes of performance evaluations used to admit students to educational programs, if variations among respondents manifest as a result of their demographic characteristics−and these variations reflect measurement confounds−then measurement invariance techniques become powerful tools for equating item content across respondents and thus improving measurement validity (e.g., Meredith, 1993; Millsap, 2011; Osterlind and Everson, 2009). Yet, in youth mental health these same demographic characteristics play important roles in explaining, for instance, the emergence of depressive symptoms in adolescence (e.g., Hankin and Abramson, 1999), disparities in access to mental health services (e.g., Barnett et al., 2021), and structural factors implicated in discrimination (e.g., Fish, 2020; Anderson et al., 2022). Under these circumstances, might users of measurement invariance techniques depress measurement validity when they seek to equate item content across measurement conditions (i.e., youth demographic characteristics) that distinguish youth for domain-relevant reasons? This question merits additional study.

In summation, the case against the use of measurement invariance techniques to detect informant discrepancies in youth mental health assessments cannot be clearer. This is an “open and shut” case (Table 1 and Figure 4A). To open this case again requires the state of the science to fundamentally “flip,” and clearly reveal that the overwhelming majority of unique variance (i.e., as reflected by informant discrepancies) produced by multi-informant assessments of youth mental health reflects measurement confounds (Figure 4B). In essence, the state of the science would need to reveal that, for decades, youth mental health researchers have been wrong to leverage data from structurally different informants, because these multi-informant assessments produce informant discrepancies that clearly do not achieve their intended purpose−to yield context-sensitive data about youth mental health. Until the state of the science “flips” in this way, it logically follows that scholars in youth mental health implement a *moratorium* on the use of measurement invariance techniques for the specific purpose of detecting informant discrepancies in youth mental health assessments. To do otherwise−to allow these techniques to continue to be applied to detect informant discrepancies−would invite empirical efforts that risk depressing the measurement validity of scores taken from instruments designed to estimate youth mental health concerns. Factors that depress measurement validity have clear, negative influences on statistical power (Markon et al., 2011), and levels of statistical power play a key role in the degree to which findings replicate across studies (Open Science Collaboration, 2015). We already have enough factors influencing “replication crises” in psychology (see Tackett et al., 2017). As a scholarly community, let us ensure that improper applications of measurement invariance techniques do not add more fuel to existing crises in confidence in psychological research findings.

## Author contributions

AD conceptualized the problem, wrote the first draft of the manuscript, and edited the manuscript. FT, AW, and GA edited the manuscript. All authors approved the manuscript and agreed to be accountable for all aspects of the work.
