# Supplementary material for: Conceptual, methodological, and measurement factors that disqualify use of measurement invariance techniques to detect informant discrepancies in youth mental health assessments
Source: Front Psychol. 2022 Aug 2;13:931296. doi: 10.3389/fpsyg.2022.931296 (PMC9378825; doi:10.3389/fpsyg.2022.931296)
Supplement: Supplementary file 1 [file Table_1.DOCX]

**Conceptual, Methodological, and Measurement Factors That
Disqualify Use of Measurement Invariance Techniques to
Detect Informant Discrepancies in
Youth Mental Health Assessments**

**Andres De Los Reyes^1*^, Fanita Tyrell^2^, Ashley L. Watts^3^, Gordon J.G. Asmundson^4^**

^1^Comprehensive Assessment and Intervention Program, University of Maryland at College Park, Department of Psychology, College Park, MD, USA

^2^Resilient Adaptation across Culture and Context Lab, University of Maryland at College Park, Department of Psychology, College Park, MD, USA
^3^University of Missouri, Department of Psychological Sciences, Columbia, MO, USA

^4^Anxiety and Illness Behaviour Laboratory, University of Regina, Department of Psychology, Regina, SK, Canada

* Correspondence:
Andres De Los Reyes ([adlr@umd.edu](mailto:adlr@umd.edu))

**ONLINE SUPPLEMENTARY MATERIAL**

**Conceptual, Methodological, and Measurement Factors That
Disqualify Use of Measurement Invariance Techniques to
Detect Informant Discrepancies in
Youth Mental Health Assessments**

**ONLINE SUPPLEMENTARY MATERIAL**

Perhaps the most often-tested hypothesis to account for the informant discrepancies observed in youth mental health assessments is the depression-distortion hypothesis (for reviews, see De Los Reyes & Kazdin, 2005; De Los Reyes et al., 2015; Richters, 1992). Briefly, the hypothesis holds that informant discrepancies stem from the fact that informants completing reports about youth (e.g., parents) often experience mental health concerns that might impact their accuracy as raters. In particular, concerns such as depressive mood are quite common among parents of youth undergoing evaluation (Goodman & Gotlib, 1999). Thus, the depression-distortion hypothesis posits that informant discrepancies are *fully* accounted for by depressed informants attending to, encoding, recalling, and rating more negative behaviors (i.e., relative to neutral or positive behaviors) in the youth being evaluated, relative to non-depressed informants rating that same youth (see also Richters, 1992).

Applications of approaches that leverage measurement invariance techniques often cite the depression-distortion hypothesis as the *primary* rationale for applying these techniques to detect informant discrepancies (e.g., Bauer et al., 2013; Clark et al., 2016; Jungersen & Lonigan, 2021; Olino et a., 2018, 2021). Yet, as we have noted elsewhere (see De Los Reyes & Makol, 2022), the depression-distortion hypothesis lies on a weak foundation of evidence. The four most important limitations of this evidence, particularly as it relates to applying measurement invariance techniques to detect informant discrepancies in youth mental health assessments, are as follows.

First, the most well-controlled investigation of the effects of depressed mood on informants’ ratings of youth behavior consists of a mood-induction study that directly compared ratings of mothers about their own children, in which mothers were randomly assigned to receive a depressed mood induction, a positive mood induction, or a neutral mood induction (Jouriles & Thompson, 1993). Following mood induction procedures, the authors had all mothers observe their child on a behavioral task and make ratings of their child’s behavior during the task. They also had independent observers rate each child’s behavior on that same task, using the same scale mothers used to rate their own child’s behavior. Mothers in the positive mood condition rated their child more positively than mothers in both the depressed mood and neutral mood conditions rated their own child. The ratings of mothers in the positive mood condition were also more positive, relative to the ratings from the independent observers. However, the authors observed null effects between mothers in the depressed mood condition and those in the neutral mood condition. The authors also observed null effects between the ratings of mothers in the depressed mood condition and the independent observers’ ratings. Taken together, experimentally inducing depressed mood among mothers did little to impact their ratings of their child’s behavior, both relative to mothers’ ratings of their own children who were assigned to a neutral mood induction, as well to independent observers’ ratings of the depression-induced mothers’ children.

Second, the most often-cited empirical support for the depression-distortion hypothesis consists of survey-based studies that tested the relations between a specific informant’s mood state (e.g., parent) as measured by self-report or survey instruments and discrepancies between that informant’s survey report of youth behavior and the survey reports of other informants about that same youth (e.g., teacher, youth; for reviews see De Los Reyes et al., 2015; De Los Reyes & Kazdin, 2005). Given the survey-based nature of these studies, even when links between informants’ mood states and informant discrepancies were detected in these studies, several parsimonious explanations account for these links. Much of this evidence might simply reflect the presence of shared informant variance between predictors and outcomes, also known as *criterion contamination* (see Garb, 2003). That is, any significant relations might reflect the “biased” informant in these studies providing self-reports for their mood state as well as reports about youth behavior used to assess informant discrepancies. Further, even if criterion contamination issues were not apparent in these studies, the depression-distortion hypothesis ignores a foundational body of work in developmental psychopathology. Specifically, youth mental health researchers rely on significant others in the lives of youth (e.g., parents and teachers) to provide reports about their behavior (De Los Reyes et al., 2022), and these informants largely account for the informants relied on in multi-informant assessments in this area of work. The mental health of these significant others has profound impacts on the development and maintenance of youth mental health concerns (e.g., Atkins et al., 2017; Goodman & Gotlib, 1999). As such, it should not come as a surprise to see a link between informants’ ratings about youth behavior and those same informants’ ratings about their own mental health. In essence, what scholars interpret as “support” for the depression-distortion hypothesis might simply reflect a reification of long-known impacts on youth mental health.

Third, if at least a portion of the links between informants’ reports and their reports about youth mental health reflect domain-relevant information (e.g., that a parent’s mental health might be contributing to the mental health concerns of the youth undergoing evaluation), it is important that studies account for this possibility. Yet, we know of no single study about the depression-distortion hypothesis that has made any attempt to decompose informants’ mood states into components that *might* reflect rater biases (e.g., difficulty concentrating) from those that *likely* reflect domain-relevant information (e.g., anhedonia, fatigue, guilt, irritability). As such, it is quite possible that the relations observed between informants’ mood states and informant discrepancies largely reflect domain-relevant phenomena.

Fourth, as we describe in detail in the main text of this paper, the last 15 years of studies informed by Achenbach and colleagues’ (1987) notion of situational specificity (i.e., the competing theory for what informant discrepancies in youth mental health assessments might reflect) accounts for each of the issues noted previously. Specifically, the body of work we review in this paper consists of (a) well-controlled laboratory studies, (b) independent assessments of domain-relevant criterion variables (i.e., that avoid criterion contamination), and (c) demonstrated links between informant discrepancies and these domain-relevant criterion variables. These studies rule out the possibility that informant discrepancies are *fully* accounted for by factors implicated in the depression-distortion hypothesis. Stated another way, the depression-distortion hypothesis posits that all informant discrepancies reflect mood-congruent rater biases. Within this lens, all the variance in informant discrepancies reflects measurement confounds—variance that is *irrelevant* to understanding youth mental health domains. In this respect, well-constructed validation studies that demonstrate a relation between informant discrepancies and domain-relevant criterion variables comprise the kind of evidence that severely diminishes the explanatory power of the depression-distortion hypothesis.

Taken together, as we argue in the main text (see Figures 1 and 2), there exist competing theories for what informant discrepancies in youth mental health assessments reflect. The existence of competing theories necessitates use of data to “settle the score.” Because competing theories exist, a full justification for applying measurement invariance techniques to detect informant discrepancies in these assessments requires data that overwhelmingly tilt in favor of these discrepancies reflecting measurement confounds. In this area, the reverse is true: The best evidence probing these discrepancies indicates that they largely contain domain-relevant information. Consequently, the weaknesses in the evidentiary base of the depression-distortion hypothesis―coupled with the strength of the body of evidence supporting situational specificity―renders this hypothesis an insufficient justification for applying measurement invariance techniques to detect informant discrepancies in youth mental health assessments.

**References Cited in Online Supplementary Material**

Achenbach, T.M., McConaughy, S.H., & Howell, C.T. (1987). Child/adolescent behavioral and emotional problems: Implications of cross-informant correlations for situational specificity. *Psychological Bulletin*, *101*(2), 213-232.
 <https://doi.org/10.1037/0033-2909.101.2.213>

Atkins, M.S., Cappella, E., Shernoff, E.S., Mehta, T.G., & Gustafson, E.L. (2017). Schooling and children's mental health: realigning resources to reduce disparities and advance public health. *Annual Review of Clinical Psychology*, *13*, 123-147.

<https://doi.org/10.1146/annurev-clinpsy-032816-045234>

Bauer, D.J., Howard, A.L., Baldasaro, R.E., Curran, P.J., Hussong, A.M., Chassin, L., & Zucker, R.A. (2013). A trifactor model for integrating ratings across multiple informants. *Psychological Methods*, *18*(4), 475-493. <https://doi.org/10.1037/a0032475>

Clark, D.A., Listro, C.J., Lo, S.L., Durbin, C.E., Donnellan, M.B., & Neppl, T.K. (2016). Measurement invariance and child temperament: An evaluation of sex and informant differences on the Child Behavior Questionnaire. *Psychological Assessment*, *28*(12), 1646-1662. <https://doi.org/10.1037/pas0000299>

De Los Reyes, A., Augenstein, T.M., Wang, M., Thomas, S.A., Drabick, D.A.G., Burgers, D., & Rabinowitz, J. (2015). The validity of the multi‐informant approach to assessing child and adolescent mental health. *Psychological Bulletin*, *141*(4), 858-900. <https://doi.org/10.1037/a0038498>

De Los Reyes, A., & Kazdin, A.E. (2005). Informant discrepancies in the assessment of childhood psychopathology: A critical review, theoretical framework, and recommendations for further study. *Psychological Bulletin, 131*(4)*,* 483-509. <https://doi.org/10.1037/0033-2909.131.4.483>

De Los Reyes, A., & Makol, B.A. (2022). Informant reports in clinical assessment. In G. Asmundson (Ed.), *Comprehensive clinical psychology*. (2^nd^ ed.). Elsevier. Advance online publication. <https://doi.org/10.1016/B978-0-12-818697-8.00113-8>

De Los Reyes, A., Talbott, E., Power, T., Michel, J., Cook, C.R., Racz, S.J., & Fitzpatrick, O. (2022). The Needs-to-Goals Gap: How informant discrepancies in youth mental health assessments impact service delivery. *Clinical Psychology Review*, *92*, 102114. <https://doi.org/10.1016/j.cpr.2021.102114>

**Garb, H.N. (2003). Incremental validity and the assessment of psychopathology in adults. *Psychological Assessment*, *15*(4), 508-520.** <http://dx.doi.org/10.1037/1040-3590.15.4.508>

Goodman, S.H., & Gotlib, I.H. (1999). Risk for psychopathology in the children of depressed mothers: A developmental model for understanding mechanisms of transmission. Psychological Review, 106(3), 458-490. 
 <https://doi.org/10.1037/0033-295X.106.3.458>

Jouriles, E.N., & Thompson, S.M. (1993). Effects of mood on mothers’ evaluations of children’s behavior. *Journal of Family Psychology, 6*(3)*,* 300-307.

<http://dx.doi.org/10.1037/0893-3200.6.3.300>

Jungersen, C.M., & Lonigan, C.J. (2021). Do parent and teacher ratings of ADHD reflect the same constructs? A measurement invariance analysis. *Journal of Psychopathology and Behavioral Assessment*, *43*(4), 778-792. <https://doi.org/10.1007/s10862-021-09874-3>

Olino, T.M., Finsaas, M., Dougherty, L.R., & Klein, D.N. (2018). Is parent–child disagreement on child anxiety explained by differences in measurement properties? An examination of measurement invariance across informants and time. *Frontiers in Psychology, 9*, 1295. <https://doi.org/10.3389/fpsyg.2018.01295>

Olino, T. M., Michelini, G., Mennies, R. J., Kotov, R., & Klein, D. N. (2021). Does maternal psychopathology bias reports of offspring symptoms? A study using moderated non‐ linear factor analysis. *Journal of Child Psychology and Psychiatry*, *62*(10), 1195-1201.

<https://doi.org/10.1111/jcpp.13394>

Richters, J.E. (1992). Depressed mothers as informants about their children: A critical review of the evidence for distortion. *Psychological* *Bulletin, 112*(3)*,* 485-499.

<https://doi.org/10.1037/0033-2909.112.3.485>
